# G6PD Polymorphisms and Hemolysis After Antimalarial Treatment With Low Single-Dose Primaquine: A Pooled Analysis of Six African Clinical Trials

**DOI:** 10.3389/fgene.2021.645688

**Published:** 2021-04-09

**Authors:** Nuno Sepúlveda, Lynn Grignard, Jonathan Curry, Laleta Mahey, Guido J. H. Bastiaens, Alfred B. Tiono, Joseph Okebe, Sam A. Coulibaly, Bronner P. Gonçalves, Muna Affara, Alphonse Ouédraogo, Edith C. Bougouma, Guillaume S. Sanou, Issa Nébié, Kjerstin Lanke, Sodiomon B. Sirima, Alassane Dicko, Umberto d’Alessandro, Taane G. Clark, Susana Campino, Ingrid Chen, Alice C. Eziefula, Roly Gosling, Teun Bousema, Chris Drakeley

**Affiliations:** ^1^Department of Infection Biology, Faculty of Infectious and Tropical Diseases, London School of Hygiene and Tropical Medicine, London, United Kingdom; ^2^CEAUL – Centro de Estatística e Aplicações da Universidade de Lisboa, Lisbon, Portugal; ^3^LGC Genomics, Hertfordshire, United Kingdom; ^4^Department of Medical Microbiology, Radboud University Medical Center, Nijmegen, Netherlands; ^5^Department of Public Health, Centre National de Recherche et de Formation sur le Paludisme & Institut National de Santé Publique, Ouagadougou, Burkina Faso; ^6^Department of International Public Health, Liverpool School of Tropical Medicine, Liverpool, United Kingdom; ^7^Department of Infectious Disease Epidemiology, Faculty of Epidemiology and Population Health, London School of Hygiene & Tropical Medicine, London, United Kingdom; ^8^MRC Unit The Gambia at the London School of Hygiene & Tropical Medicine, Fajara, Gambia; ^9^Malaria Research and Training Centre, Faculty of Pharmacy and Faculty of Medicine and Dentistry, University of Sciences, Techniques and Technology of Bamako, Bamako, Mali; ^10^Department of Disease Control, Faculty of Infectious and Tropical Diseases, London School of Hygiene & Tropical Medicine, London, United Kingdom; ^11^Department of Epidemiology and Biostatistics, University of California, San Francisco, San Francisco, CA, United States; ^12^Department of Global Health and Infection, Brighton and Sussex Medical School, University of Sussex, Brighton, United Kingdom

**Keywords:** genetic association study, clinical trials, malaria, drug safety, hemoglobin

## Abstract

Primaquine (PQ) is an antimalarial drug with the potential to reduce malaria transmission due to its capacity to clear mature *Plasmodium falciparum* gametocytes in the human host. However, the large-scale roll-out of PQ has to be counterbalanced by the additional risk of drug-induced hemolysis in individuals suffering from Glucose-6-phospate dehydrogenase (G6PD) deficiency, a genetic condition determined by polymorphisms on the X-linked *G6PD* gene. Most studies on G6PD deficiency and PQ-associated hemolysis focused on the G6PD A- variant, a combination of the two single nucleotide changes G202A (rs1050828) and A376G (rs1050829), although other polymorphisms may play a role. In this study, we tested the association of 20 G6PD single nucleotide polymorphisms (SNPs) with hemolysis measured seven days after low single dose of PQ given at the dose of 0.1 mg/kg to 0.75 mg/kg in 957 individuals from 6 previously published clinical trials investigating the safety and efficacy of this drug spanning five African countries. After adjusting for inter-study effects, age, gender, baseline hemoglobin level, PQ dose, and parasitemia at screening, our analysis showed putative association signals from the common G6PD mutation, A376G [−log_10_(*p*-value) = 2.44] and two less-known SNPs, rs2230037 [−log_10_(*p*-value] = 2.60), and rs28470352 [−log_10_(*p*-value) = 2.15]; A376G and rs2230037 were in very strong linkage disequilibrium with each other (*R*^2^ = 0.978). However, when the effects of these SNPs were included in the same regression model, the subsequent associations were in the borderline of statistical significance. In conclusion, whilst a role for the A- variant is well established, we did not observe an important additional role for other G6PD polymorphisms in determining post-treatment hemolysis in individuals treated with low single-dose PQ.

## Introduction

The last two decades of intensive malaria control have achieved a significant reduction of malaria cases and deaths and, consequently, several endemic countries were officially declared in either pre-elimination, elimination, or eradication stages. One approach to accelerate disease elimination is to target the sexual and transmission stages of the parasite and, thereby, block transmission from humans to mosquitoes. The 8-aminoquinoline, Primaquine (PQ), can clear *P. falciparum* gametocytes and is typically used as a single-dose treatment. The drug at larger doses has also the ability to clear *Plasmodium vivax* and *Plasmodium ovale* hypnozoites. However, the biochemical action of the drug is known to induce transient hemolysis in treated individuals. The recognition of this side effect led to the evaluation of the efficacy and safety of treatments based on lower single doses of PQ for gametocyte clearance in 2011 (Eziefula et al., 2014). Later, the World Health Organisation (WHO) revised treatment guidelines and reduced the recommended PQ dose from 0.75 to 0.25 mg/kg in areas of *P. falciparum* elimination and/or high malaria drug resistance (WHO Malaria Policy Advisory Committee and Secretariat, 2012; World Health Organization, 2012). After this revision, several randomized controlled clinical trials and community studies were performed with the specific aim to assess both the efficacy and the safety of different PQ drug regimens, including the one based on the new recommended dose (Dicko et al., 2016; Gonçalves et al., 2016; Stone et al., 2017; Bastiaens et al., 2018; Chen et al., 2018).

The potential use of PQ-based treatments in large-scale malaria control and elimination campaigns might pose an additional risk to individuals with Glucose-6-phospate dehydrogenase (G6PD) deficiency (Baird and Surjadjaja, 2011; Awandu et al., 2018; Chen et al., 2018), a common red blood cell disorder in Africa (Howes et al., 2012). G6PD is a key enzyme in the pentose phosphate pathway that controls oxidative damage in erythrocytes. It is encoded by the *G6PD* gene, which is located in the telomeric region of the long arm of the X chromosome (Xq28). Since it results from an X-linked recessive transmission, G6PD deficiency is more frequent in males but effects are more difficult to predict in females due to random X-chromosome inactivation. More than 150 single nucleotide polymorphisms (SNPs) have been identified in this gene with different impact on hemolysis and activity of the enzyme (Clarke et al., 2017). Additionally, there are specific variants that tend to confer protection against severe malaria-related anemia or cerebral malaria and these variants are under natural selection in malaria endemic countries (Maiga et al., 2014; Manjurano et al., 2015; Clarke et al., 2017; Sepúlveda et al., 2017). In Africa, the most important genetic markers for G6PD deficiency are the single nucleotide polymorphism (SNP) rs1050828 (G202A, or C202T, chrX:154,536,002) and rs1050829 (A376G, or T376C, chrX:154,535,277) (Rockett et al., 2014). A combination of these two single nucleotide changes (202A and 376G), also known as the A- variant, is associated with an 88% loss of G6PD enzyme activity (Hirono and Beutler, 1988; Beutler et al., 1989), which decreases the tolerance to 8-aminoquinolines. In principle, G6PD polymorphisms other than the classical A- variant should not be associated with significant hemolysis after a single-dose PQ treatment, but this expectation has never been tested with data from the field.

To fill in this research gap, we performed a genetic association analysis of 20 G6PD SNPs using data of 952 individuals from 6 PQ-related clinical trials in Africa: 3 dose-efficacy trials and 3 safety trials. These clinical trials were mostly conducted in G6PD-normal individuals. The subsequent findings aimed to contribute to a more accurate pharmacovigilance of PQ-treatment in the continent.

## Materials and Methods

### Outline of Available PQ Clinical Trials

Data from 957 individuals enrolled in six PQ clinical trials in Africa were made available for this analysis (Table 1): 330, 107, and 367 individuals from dose-efficacy studies performed in Burkina Faso (BF1), Kenya (KEN), and Uganda (UGD), respectively (Eziefula et al., 2014; Gonçalves et al., 2016; Stone et al., 2017); 77, 50, and 26 individuals from safety studies conducted in Burkina Faso (BF2), The Gambia (GAM), and Mali (MAL), respectively (Dicko et al., 2016; Bastiaens et al., 2018).

**TABLE 1:** Demographics of each PQ safety and dose-efficacy studies.

Characteristics	BF1	BF2	GAM	KEN	MAL	UGD
Reference	Gonçalves et al., 2016	Bastiaens et al., 2018	Bastiaens et al., 2018	Stone et al., 2017	Dicko et al., 2016	Eziefula et al., 2014
Type of study	Dose-efficacy	Safety	Safety	Dose-efficacy	Safety	Dose-efficacy
Sample size	330	77	50	107	26	367
G6PD status	BinaxNOW RDT	Beutler’s FST	Beutler’s FST	N/A	Beutler’s FST	Beutler’s FST
Males (n,%)	170 (51.5)	77 (100)	50 (100)	59 (55.1)	26 (100)	183 (49.9)
Mean age (range)	7.7 (2-14)	28.8 (18-44)	16.7 (10-40)	9.6 (5-15)	35.3 (18-50)	5.1 (1-10)
**Primaquine dose (n,%)**						
0.00 (placebo)	105 (31.8)	10 (13.0)	0 (0.0)	53 (49.5)	0 (0.0)	74 (20.2)
0.10-0.40 mg/kg	225 (68.2)	67 (87.0)	50 (100.0)	54 (50.5)	5 (19.2)	201 (54.8)
0.41-0.75 mg/kg	0 (0.0)	0 (0.0)	0 (0.0)	0 (0.0)	21 (80.8)	92 (25.1)
**Mean parasitemia (range, parasites/μl)**						
Day 0	5984.5 (0-237986)	373.8 (0-3348)	90.2 (0-2384)	1692.2 (0-31360)	0 (0)	69766.5 (48-518180)
Day 7	0.4 (0-124)	0 (0-0)	0 (0-0)	1.6 (0-40)	0 (0)	0 (0-16)
**Mean hemoglobin (range, g/dL)**						
Day 0	11.5 (6-14.3)	14.2 (11.4-17.5)	13.1 (11-17.2)	12.0 (9.6-14.8)	15.0 (13.3-17.5)	11.2 (8-15.4)
Day 7	11.3 (7.9-15.1)	13.5 (10.22-17.1)	12.5 (9.9-17.5)	12.2 (8.6-14.8)	14.3 (12-16.2)	10.8 (7.4-15.5)

Briefly, only children were recruited in the BF1, KEN, and UGD dose-efficacy studies. With respect to the safety studies, only male adults were enrolled in BF2 and MAL whilst male adolescents were the majority of the participants from the GAM study. Only G6PD-normal individuals were considered eligible to participate in BF1, MAL, and UGD. In contrast, BF2 and GAM included both deficient and normal individuals with respect to G6PD enzymatic activity. In KEN, the G6PD status was not screened at enrollment. In all safety studies, PQ treatment was administrated together with a partner drug at day 0 of the trial. In the dose-efficacy studies, participants received a 3-day course of dihydroartemisinin-piperaquine (DP) alone or with a single low dose of PQ (0.25 mg/kg) on the third day of DP treatment (day 2 of the trial). For additional information about participant’s recruitment, inclusion/exclusion criteria, and ethics, consult the original research protocol of each study.

### Measurement of Hemoglobin Levels and Parasitemia

At enrollment (day 0) and on day 7 of the trials, all study participants had their hemoglobin (Hb) concentration quantified (g/dL) and had a blood slide prepared to detect malaria parasites in 100 microscopic fields and quantified against 200 and 500 leukocytes, and then finally translated into parasite counts/μl under the assumption of 8,000 leukocytes per μl.

### Diagnostics of G6PD Deficiency

Participants were screened for G6PD deficiency at enrollment in all studies with the exception of KEN. The BinaxNOW rapid diagnostic test (RDT; Alere Inc., Waltham, MA, United States) was used in BF1 and Beutler’s fluorescence spot test (FST R&D Diagnostics, Greece) was used in all other studies where G6PD deficiency was screened.

### DNA Extraction and Genotyping

DNA was extracted from dried blood spots (DBS) from UGD, KEN, and BF1 using the QIAamp DNA Mini Kit (Qiagen, United Kingdom). DNA was extracted from whole blood samples from GAM, MAL, and BF2 using the QIAamp DNA Blood Mini Kit (Qiagen, United Kingdom). All extractions were performed according to manufacturer’s recommendations.

SNPs were genotyped using either the Kompetitive Allele Specific PCR (KASP) assay (LGC Genomics, United Kingdom) or a customized version of the High-throughput Mutation Screen kit for G6PD (Diacurate Inc., China). The KASP assay (Panel 1: GAM and BF1 and Panels 2: BF2, Supplementary Table 1) contains two allele-specific forward primers, one common reverse primer specific for each SNP, 0.8 μL of genomic DNA suspension and 0.8 μL 2 × KASP reaction mix. The High-throughput Mutation Screen kit for G6PD consists of a multiplex extension and ligation-based probe amplification directly from DBS and was performed in China by Diacurate Incorporated. The customized assay interrogates 23 SNPs (Panel 3: KEN, MAL, and UGD, Supplementary Table 1). Following completion of the PCR, each sample was assigned a genotype based on cluster plot analysis of raw data using LGC’s proprietary Kraken software.

### Genetic Data and Quality Controls

To ensure high quality SNP data, one needs to perform routine quality checks for genetic association studies (Grabowska et al., 2020). In this regard, we excluded all SNPs from the analysis if these SNPs were monomorphic (three SNPs), had a minor allele frequency <5% (seven SNPs), had a heterozygous frequency >2% on males (one SNP), had a strong deviation from the Hardy-Weinberg equilibrium on females (two SNPs; *p* < 0.001; Chi-square goodness-of-fit tests), and had a frequency of missing data >10% (thirteen SNPs). After these quality checks, there was evidence for a total of 20 high-quality SNPs to be used in the subsequent genetic association analysis. For a matter of comparison, allelic frequencies of African populations (*n* = 1,003 alleles in total) were compiled from the 1000 Genome Project as available in the database dbSNP (Auton et al., 2015). All of these genetic markers were annotated according to the forward strand and the reference genome assembly GRCh38.

### Genetic Association Analysis

A linear regression approach was applied to study the genetic association between the selected SNPs and the hemoglobin level measured on day 7 after PQ treatment. For statistical convenience, the hemoglobin level was log-transformed so that the resulting data would follow an approximate Normal distribution. The statistical assessment of a genetic association between a particular SNP and the phenotype of interest was based on the comparison between two nested regression models using Wilk’s log-likelihood ratio test. The first model was a non-genetic linear regression model that included the following covariates: study index, gender, age, presentation parasitemia, and hemoglobin level, both day on presentation (hereafter considered as day 0). The second model is a genetic linear regression that extends the latter by additionally including a given genetic effect of SNP under analysis. For each SNP, five different genetic effects were included in the analysis: general effect (no specific parametric structure on the genotype effects), dominance/recessive (genotypes including the dominant allele had the same effect), additive (the effect of an individual specific allele in the outcome is additive), and heterosis (both homozygous genotypes have the same effect). To simplify the presentation of the results, statistical significance for the association between a given SNP and the outcome was assessed by the minimum *p*-value of all the five *p*-values comparing the non-genetic model and five genetic models for the SNP under analysis. To control for multiple testing, the Benjamini-Hochberg procedure was applied to all the minimum *p*-values in order to obtain an overall false discovery rate of 5% (Benjamini and Hochberg, 1995). This procedure was based on the following algorithm: (i) order the *p*-values from the largest to the smallest, (ii) calculate the largest k that satisfy *p_*k*_* < 0.05/*k* where p_*k*_ is the *k*-th ordered *p*-value; (iii) reject the null hypothesis for all ordered *p*-values lower than *p*_*k*_. Statistically significant SNPs were then assessed in terms of their linkage disequilibrium (LD) using the correlation coefficient R^2^.

## Results

### Inter-Study Variations in Hemoglobin Levels and PQ Treatment Doses

This study encompassed a total of 957 individuals distributed across six studies with different sample sizes (Table 1). With respect to PQ treatment, BF1, BF2, and KEN studies compared a placebo group to another group treated with a PQ dose between 0.1 mg/kg and 0.4 mg/kg. The GAM and MAL studies only included a single treatment group with a 0.1-0.5 mg/kg PQ dose. The study from Uganda was the only one encompassing three treatment arms (placebo, 0.1-0.4 mg/kg and 0.41-0.75 mg/kg). In MAL, only *P. falciparum* negative individuals were recruited to the study.

**FIGURE 1:**
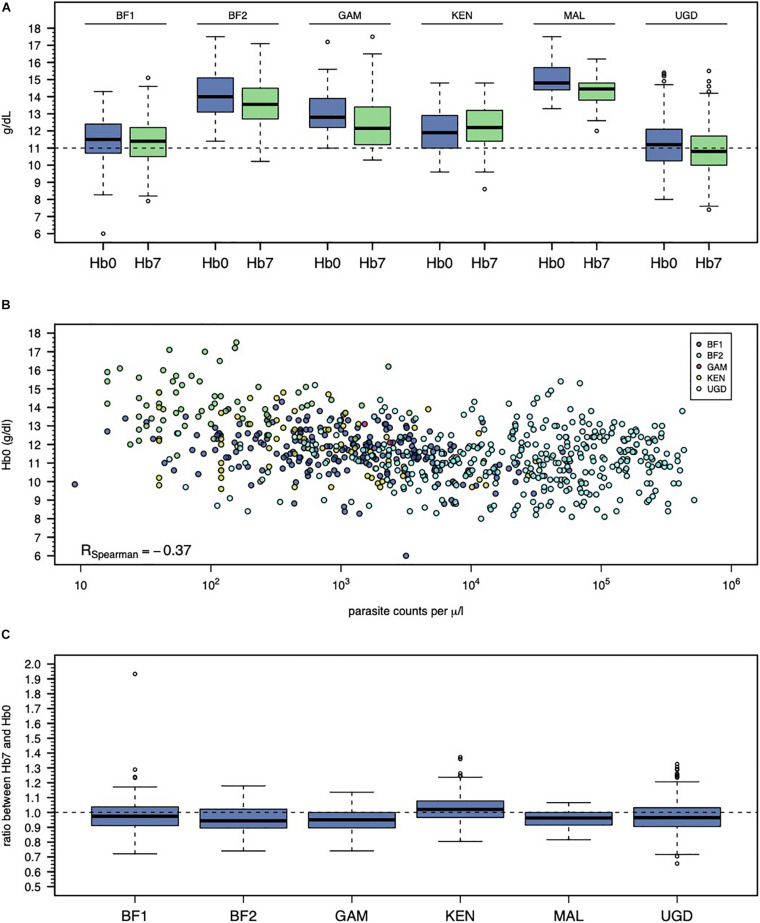
Inter-study variation of Hb0 and Hb7. **(A)** Violin plots of Hb0 per study. **(B)** Relationship between Hb0 and parasitemia in infected individuals. Note that the study from Mali is not shown because it only recruited non-infected individuals. **(C)** Violin plots of the ratio between Hb0 and Hb7.

On the day of treatment (day 0), the mean Hb ranged from 11.2 to 15.0 (overall range = 6.0-17.5) g/dL (Table 1). Since the targeted populations and recruitment strategies differed across studies (*e.g.*, male adults in MAL study versus male and female children in UGD study), an inter-study variation on mean (or median) Hb levels on day 0 was expected (Figure 1A). Another possible source of inter-study variation on Hb levels on day 0 (Hb0) was related to differences in parasitemia at presentation (Figure 1B). Hb0 was lower with the higher parasite counts in the blood (Spearman’s correlation coefficient = −0.37, *p* < 0.001). Unsurprisingly, PQ treatment was associated with a drop in the Hb levels at day 7 (Hb7) in the majority of the individuals (Figure 1C) with the level of reduction differing by study. The studies showed substantial heterogeneity in Hb levels, both on days 0 and 7 after PQ treatment.

When a non-genetic linear regression model was initially fitted to the data (Supplementary Table 2), there was evidence for a negative effect of parasitemia, a positive effect of age, and a positive effect of log-Hb0 on the log-Hb7 coefficient estimates = −0.033, 0.004, and 0.567, respectively, all *p*-values <0.001). In addition, the effects of different studies on log-Hb7 were not statistically significant (*p* > 0.05) with the exception of the study from Kenya (coefficient estimate = 0.047, *p* < 0.001; Figure 1C). The effect of each PQ dose range was negative on the Hb levels at day 7 (coefficient estimates = −0.010 and −0.019 for 0.1–0.4 and >0.4 mg/kg, respectively), which suggested some degree of hemolysis due to PQ treatment itself. However, these effects referring to PQ dose ranges were not statistically significant (*p*-values = 0.195 and 0.112, respectively). Finally, this initial model including study index, gender, age, parasitemia at presentation, and Hb0 as covariates could explain 61% of the total variation observed in the log-Hb7 levels (adjusted *R*^2^ = 0.61). We then investigated whether genetic variation on *G6PD* locus could explain the remaining random variation in the outcome.

### Analysis of *G6PD* SNPs

Genetic analysis initiated by assessing the quality of the genotype data. Twenty out of the 23 initially genotyped SNPs were considered with high quality and, thus, they were carried forward to the association study (Supplementary Table 3). Of note, ten of the selected SNPs were located within the coding region of the *G6PD* gene. Two of these SNPs represented synonymous variants, other two referred to known missense variants with pathogenic potential (rs1050829 and rs1050828) and the remaining six SNPs were variants in the intronic regions of the *G6PD* gene. The other 10 SNPs were located in the flanking regions of *G6PD*, namely, in intronic regions of *TLK1*, *RPL10*, *SNORA70*, and *IKBKG* genes. Twelve of these SNPs could not be genotyped in the samples from BF2 due to unavailability of DNA material to genotype these SNPs.

**FIGURE 2:**
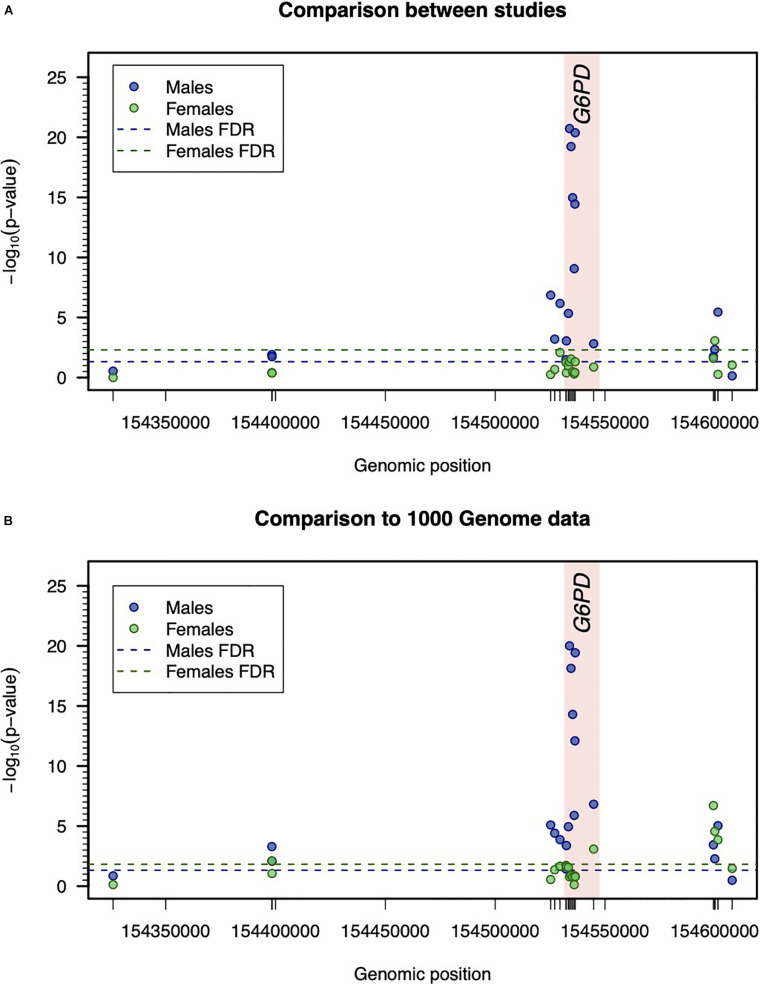
Inter-study variation with respect to the genetic data. **(A)** Comparison of genotype frequencies between studies. Statistical significance was determined by –log_10_ (*p*-value) associated with the Pearson’s test for independence in two-way frequency tables. For males and females, the thresholds for statistical significance were calculated separately and were determined to ensure a false discovery rate (FDR) of 5%. **(B)** Comparison between allelic frequencies between studies and 1000 Genome Project data. The reported –log_10_ (*p*-value) refers to the minimum *p*-value of Binomial test for comparing the allelic frequency of a given SNP in each study assuming the respective allelic frequency of 1000G project data as the one under the null hypothesis (see Table 2).

In male participants, the allele frequency profile substantially differed from one study to another, specifically, in SNPs located within or very close to the coding region of *G6PD* (Table 2 and Figure 2A). The same finding was obtained when the allele frequencies associated with each study were compared to those available from the combined 1000 Genome Project for African populations (Figure 2B and Supplementary Figure 1). Interestingly, this variation became less obvious in SNPs located in the respective flanking regions, probably due to a decrease in linkage with variants within the *G6PD* locus.

**TABLE 2:** Frequencies of the reference alleles associated with each of the 20 SNPs located in the *G6PD* locus defined between 154,326,058 and 154,607,918 positions of X chromosome where the coding region of the gene is located between 154,531,390 and 154,547,569.

SNP (Associated Gene)	Position	Functional consequence	Ref > Alt Allele	Gender	BF1	BF2	GAM	KEN	MAL	UGD	1000G
rs766420 (*TKL1*)	154,326,058	Intron	C > G	Male	0.355	N/A	0.440	0.460	0.269	0.330	0.365
				Female	0.359	N/A	N/A	0.344	N/A	0.361	
rs915941	154,398,308	Upstream	A > C	Male	0.591	N/A	0.480	0.524	0.231	0.554	0.426
(*RPL10,SNORA70*)		transcription variant		Female	0.500	N/A	N/A	0.562	N/A	0.535	
rs915942	154,398,397	Upstream	G > A	Male	0.526	N/A	0.667	0.607	0.846	0.606	0.588
(*RPL10,SNORA70*)		transcription variant		Female	0.635	N/A	N/A	0.562	N/A	0.584	
rs28470352 (N/A)	154,525,272	N/A	T > A	Male	0.702	N/A	0.420	0.629	0.231	0.722	0.663
				Female	0.650	N/A	N/A	0.719	N/A	0.671	
rs61042368 (N/A)	154,527,122	N/A	G > A	Male	0.751	N/A	0.960	0.885	0.926	0.840	0.873
				Female	0.834	N/A	N/A	0.885	N/A	0.894	
rs12389569 (N/A)	154,529,519	N/A	G > A	Male	0.900	N/A	0.714	0.902	0.808	0.978	0.903
				Female	0.886	N/A	N/A	0.958	N/A	0.938	
rs77214077	154,532,214	Coding	G > A	Male	0.953	N/A	0.960	0.903	0.923	0.861	0.910
(*G6PD*)		Synonymous		Female	0.947	N/A	N/A	0.854	N/A	0.902	
rs2230037 (*G6PD*)	154,532,439	Coding,	G > A	Male	0.674	0.861	0.800	0.661	0.962	0.766	0.714
		Synonymous		Female	0.667	N/A	N/A	0.698	N/A	0.660	
rs73573478	154,533,349	Intron	G > A	Male	0.749	0.987	0.960	0.885	0.962	0.835	0.875
(*G6PD*)				Female	0.833	N/A	N/A	0.875	N/A	0.894	
rs2515905 (*G6PD*)	154,533,860	Intron	G > A	Male	0.888	0.351	0.780	0.839	0.481	0.833	0.827
				Female	0.838	N/A	N/A	0.833	N/A	0.799	
rs2515904 (*G6PD*)	154,534,556	Intron (pathogenic)	G > C	Male	0.889	0.377	0.780	0.836	0.462	0.833	0.827
				Female	0.838	N/A	N/A	0.833	N/A	0.796	
rs1050829^a,b^	154,535,277	Missense variant	T > C	Male	0.694	0.234	0.420	0.629	0.192	0.722	0.662
(*G6PD*)				Female	0.625	N/A	N/A	0.719	N/A	0.668	
rs1050828^c^ (*G6PD*)	154,536,002	Missense variant	C > T	Male	0.930	1.000	0.804	0.885	0.462	0.887	0.865
				Female	0.870	N/A	N/A	0.854	N/A	0.872	
rs762515 (*G6PD*)	154,536,313	Intron	T > C	Male	0.684	0.227	0.420	0.629	0.154	0.695	0.634
				Female	0.619	N/A	N/A	0.708	N/A	0.655	
rs762516 (*G6PD*)	154,536,448	Intron	C > T	Male	0.888	0.355	0.780	0.836	0.462	0.833	0.827
				Female	0.838	N/A	N/A	0.833	N/A	0.799	
rs113492957	154,544,847	Intron	C/T	Male	0.756	N/A	0.960	0.885	0.962	0.846	0.898
(*G6PD,IKBKG*)				Female	0.838	N/A	N/A	0.865	N/A	0.894	
rs4898389^b^ (N/A)	154,599,376	N/A	G > A	Male	0.977	N/A	0.900	0.952	0.962	0.897	0.904
				Female	0.978	N/A	N/A	0.906	N/A	0.935	
rs5986877^b^ (N/A)	154,600,008	N/A	G > C	Male	0.982	N/A	0.960	0.952	0.962	0.892	0.933
				Female	0.984	N/A	N/A	0.896	N/A	0.935	
rs7879049^b^ (N/A)	154,601,444	N/A	G > A	Male	0.497	N/A	0.800	0.742	0.846	0.594	0.663
				Female	0.560	N/A	N/A	0.604	N/A	0.614	
rs60030796 (N/A)	154,607,918	N/A	A > G	Male	0.901	N/A	0.960	0.919	0.885	0.914	0.907
				Female	0.900	N/A	N/A	0.969	N/A	0.938	

*The reference (Ref) and alternative (Alt) alleles are given in relation to the minus strand. The last column refers to allelic frequency as reported in the combined 1000 genome project (1000G) for African populations.^*a*^ T376C or equivalently A376G. ^*b*^ Triallelic or quadriallelic SNPs according to reference genome assembly GRCh38. ^*c*^ C202T or equivalently G202A.*

In female participants, the allele frequency profile did not significantly differ between studies (Figure 2A). Importantly, the allelic frequency profile was consistent with the one available from the combined 1000 Genome project for African populations (Figure 2B).

The wild type allele of G6PD-deficiency SNP (G202A, rs1050828) was found at a high frequency in most of the studies. The respective relative frequency ranged from 46.2% (MAL) to 100% (BF2) in male participants, whilst it was approximately constant in female participants (from 85.4% in KEN to 87.2% in UGD). These results are consistent with only recruiting participants with normal G6PD enzymatic activity in most of the studies and, therefore, the respective data was expected to be enriched with wild type alleles of rs1050828.

### Association Analysis Between *G6PD* SNPs and Hemolysis After PQ Treatment

**FIGURE 3:**
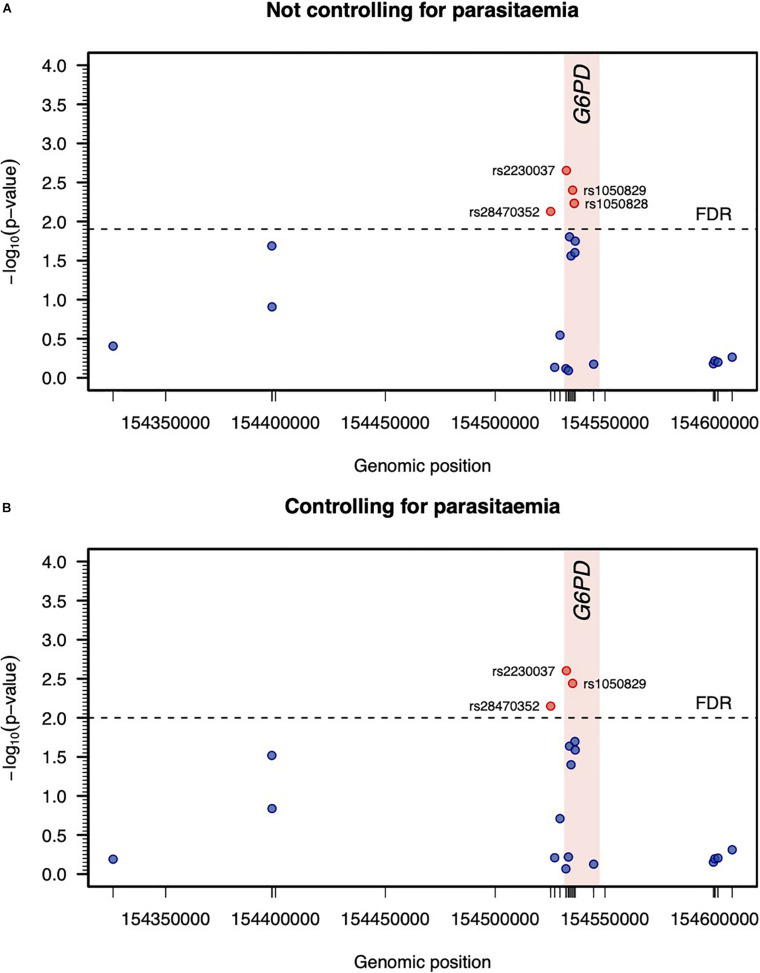
Genetic association analysis between log-Hb7 and each SNP adjusting or not for parasitemia at day 0 (**A,B**, respectively). Statistical significance was determined by the maximum of −log_10_ (*p*-value) associated with the log-likelihood ratio tests when testing different genetic effects of each SNP in a linear regression model adjusting for age, gender, parasitemia at screening, and log-Hb0. The threshold for statistical significance was determined to ensure a false discovery rate (FDR) of 5%.

Since MAL did not include infected individuals, two separate genetic association analyses related to variation of log-Hb7 were conducted adjusting for previous confounders but including or not parasitemia at screening as an additional covariate. The strength of association for each SNP was almost indistinguishable adjusting or not for parasitemia. In particular, both analyses suggested putative associations of rs28470352, rs2230037, and rs1050829 (A376G) with log-Hb7 (Figures 3A,B). There was evidence for a very strong LD between rs2230037 and rs1050829 (*R*^2^ = 0.978). In contrast, these SNPs were in weak LD with rs28470352 (*R*^2^ = 0.208 and 0.227, respectively). These three polymorphisms were then carried forward to the final stage of the analysis. There was an additional association between rs1050828 (G202A) with the phenotype that was only statistically significant adjusting for confounding but excluding parasitemia (Figure 3A).

The final stage of the association analysis comprised the assessment of the joint effect of the three detected SNPs on the phenotype using extended regression models where genetic and non-genetic effects were contemplated (Table 3). Since there were no data available for rs28470352 from BF2, two extended models were fitted to the data including or not the effect of that genetic marker; these models were denoted hereafter as M_1_ and M_2_, respectively. As a consequence, the estimation of M_1_ was based on a smaller sample size (*n* = 880) due to the exclusion of the data from that SNP. In contrast, the estimation of M_2_ was based on the whole data set (*n* = 957).

**TABLE 3:** Estimation of the joint genetic models M_1_ (excluding data from BF2) and M_2_ (based on the whole data set) for the association between log-Hb7 and the best *G6PD* SNP adjusting or not for parasitemia at day 0 where the alleles A and B denote the reference and alternative alleles shown in Table 2.

	Model M_1_		Model M_2_	
Predictor	Estimate (SE)	*P*-value	Estimate (SE)	*P*-value
Intercept	1.021 (0.068)	<0.001	1.020 (0.066)	<0.001
**Study**				
BF1 (reference)	–	–	–	–
BF2	NA (NA)	NA	−0.015 (0.021)	0.463
GAM	−0.019 (0.019)	0.327	−0.014 (0.019)	0.442
KEN	0.047 (0.011)	<0.001	0.047 (0.011)	<0.001
MAL	−0.018 (0.033)	0.577	−0.004 (0.029)	0.903
UGD	0.015 (0.009)	0.100	0.012 (0.009)	0.159
Age (per year)	0.005 (0.001)	<0.001	0.004 (0.001)	<0.001
**PQ dose (mg/kg)**				
0	–	–	–	–
0.10-0.40	−0.007 (0.008)	0.399	−0.007 (0.008)	0.328
0.41-0.75	−0.016 (0.012)	0.175	−0.017 (0.012)	0.159
**Gender**				
Female (reference)	–	–	–	–
Male	−0.010 (0.009)	0.261	−0.012 (0.009)	0.193
log-Hb0	0.561 (0.029)	<0.001	0.565 (0.027)	<0.001
Parasitemia (x 100,000)	−0.033 (0.005)	<0.001	−0.033 (0.005)	<0.001
**rs28470352**				
TT (reference)	–	–	–	–
TA	−0.003 (0.040)	0.946	–	–
AA	−0.061 (0.065)	0.353	–	–
**rs2230037**				
GG (reference)	–	–	–	–
GA	0.007 (0.010)	0.467	0.006 (0.010)	0.506
AA	0.020 (0.010)	0.041	0.018 (0.009)	0.051
**rs1050829**				
TT (reference)	–	–	–	–
CT	−0.012 (0.040)	0.768	−0.016 (0.010)	0.117
CC	0.051 (0.065)	0.439	−0.011 (0.009)	0.208

Both M_1_ and M_2_ led to estimates for the non-genetic variables similar to the ones obtained in absence of genetic effects (Table 3). More importantly, the effects of rs28470352 and rs1050829 were not statistically significant. Positive effects on log-Hb7 were observed for the AA genotype of rs2230037 (coefficient estimates = 0.020 and 0.018 for M_1_ and M_2_, respectively). When transformed into linear scale, these effects predicted an increase of 1.02 g/dL in Hb7 when compared to the GG genotype of rs223037. However, these effects were in the borderline of statistical significance (*p*-values = 0.041 and 0.051, respectively). In conclusion, the effect size and its statistical significance suggested that these genetic markers appear to have a minor role in explaining Hb7 in relation to non-genetic factors, such as age, parasitemia or Hb0.

## Discussion

This study aimed to evaluate the effect of genetic polymorphisms within *G6PD* locus on hemolysis on individuals treated with single doses of PQ. The rationale was to provide information for safety concerns associated with the broader population delivery of PQ given the potentially high prevalence of G6PD deficiency in African populations. This was an opportunistic study, typing *G6PD* SNPs in retrospective samples collected from completed efficacy or safety of single dose PQ in which Hb data were collected and, as such, there is a number of limitations. Notwithstanding this fact, we identified putative associations of the common A376G variant in African population and two less-known SNP with Hb7. However, these *G6PD* polymorphisms appear to have minor effects when compared to the strong non-genetic effects related to age, parasitemia, and Hb0. This finding is reassuring from the point of drug safety, because it seems to rule out any specific effect of *G6PD* variants on hemolysis other than the PQ effect alone. In addition, it is important to note that, as described in the original papers, hemolysis was only transient in the study participants who had normal levels of Hb at the end of the respective trials. Therefore, the drug could be considered safe irrespective of the *G6PD* variation present in the study participants, as discussed in the original references (Bastiaens et al., 2018; Chen et al., 2018) and in a safety study from Tanzania (Mwaiswelo et al., 2016).

As stated above a limitation is that five out of six studies in our analysis excluded individuals with reduced G6PD enzymatic activity and, therefore, the deleterious allele A in G202A was under-represented in the data through selection bias. This exclusion criterium reduced the probability to detect any putative association between that SNP and Hb7. Previous associations with drops in Hb and both G202A and A376G have been shown in treatment trials with PQ but this was with the higher dose of 0.75 mg/kg (Shekalaghe et al., 2009, 2010). Interestingly, the one individual in those studies that had a significant drop in Hb levels was wild type at both SNPs suggesting involvement of other genes elsewhere in the genome, which was in part the motivation for this study.

We addressed further differences between studies by adjusting for age and gender in our genetic association analysis. In addition, age and gender could also control for possible confounding on the dynamics of Hb during treatment and these were assumed to be independent of other variables analyzed. A study-specific effect was also included in the linear regression models to capture latent (genetic) differences between populations and potential differences in research protocols. Interestingly, all studies but KEN had similar study-specific effects in the data. This suggests that the level of hemolysis observed at day 7 was fairly consistent across studies. These study-specific effects together with the effects of parasitemia and Hb0 explained more than half of the variation observed in Hb7 and suggested that the remaining variation is not explained by the genetic variation in the *G6PD* locus. Further variation might be due to further individual variation in the rate by which the drug is metabolized in the body. Genetic polymorphisms on *CYP2D6* gene, which are known to influence the rate by which PQ is metabolized in the body (Gonçalves et al., 2017; Saito et al., 2018), have been associated with variation in post treatment gametocyte carriage using data from 8 clinical trials including the ones here analyzed (Pett et al., 2019), but a high proportion of missing *CYP2D6* data precluded a more integrated genetic association analysis including the joint effects of *G6PD* and *CYP2D6* allelic variants on hemolysis.

The KEN study was identified as having a higher study-specific effect than the remaining studies. This study was the only one where G6PD deficiency status was not determined in the participants at enrollment (Stone et al., 2017). However, the frequency of the deleterious allele A associated with G202A was consistent with the other studies.

Our study comprised a relatively small number of genotyped SNPs when compared to more than 250 genetic variants already identified within the *G6PD* locus (Clarke et al., 2017). Despite this hypothetical large number of genetic variants in the *G6PD* locus, many of catalogued SNPs have too-low minor allele or they are only present in non-African populations. In this regard, the evaluated SNPs were considered the most informative in terms of allele frequency in African populations. As a limitation, half of the evaluated SNPs were outside the *G6PD* coding region. Given this scenario, it is then possible that additional association signals could derived from rare genetic variants located in this locus. However, these association signals could only be detected in studies with large sample sizes, as illustrated by the UK10k consortium (Walter et al., 2015) and the study of genetic variants related to blood pressure (Surendran et al., 2020). It is worth noting that the telomeric location of the *G6PD* gene on the X chromosome imposes serious limitations in terms of the assay costs and scalability to a large number of samples and SNP under analysis, as illustrated in the most comprehensive genetic study of *G6PD* variation conducted so far (Clarke et al., 2017). From an initial number of more than 250 SNPs, the authors considered the study only feasible in a benchmark set of 65 SNPs located in a 560 kb region spanning the *G6PD* locus itself and part of the overlapping *IKBKB* gene (encoding Inhibitor of Kappa Light Polypeptide Gene Enhancer in B-Cells). Since our study comprised the analysis of retrospective data, there was a limitation to the number of SNP under analysis. In this regard, we considered these SNPs to be a good representation of this reference set, because only three SNPs were discarded after our quality control checks.

Despite the inherent limitations in attempting to combine different studies, all used a single dose of PQ, had measures of Hb7 after the initiation of treatment and the same core group of G6PD-associated SNPs were tested. Consistent with previous reports we reported borderline associations of specific *G6PD* SNPs with mild transient hemolysis. This provides further evidence that single, low dose PQ can be deployed in larger population studies.

## Data Availability Statement

The raw data supporting the conclusion of this article will be made available by the authors, without undue reservation.

## Ethics Statement

The studies involving human participants were reviewed and approved by the Ethics Committee of the Faculty of Medicine, Pharmacy, and Dentistry, University of Science, Techniques and Technologies of Bamako, and the Committee on Human Research at the University of California, San Francisco (studies in Mali); the Comité d’Ethique pour la Recherche en Santé, Ministère de la Santé du Burkina Faso, and the Comité Technique d’Examen des Demandes d’Autorisation d’Essais Cliniques, Ministère de la Santé du Burkina Faso (studies in Burkina Faso); the Gambia Government/MRC Joint Ethics Committee (studies in the Gambia); the Makerere University School of Medicine Research Ethics Committee and the Uganda National Council of Science and Technology (study in Uganda); the Kenya Medical Research Institute Ethics Review Committee (study in Kenya); and the Interventions Research Ethics Committee of the London School of Hygiene and Tropical Medicine (all studies). Written informed consent to participate in this study was provided by the participants’ legal guardian/next of kin.

## Author Contributions

TB and CD conceived the research. JC, LM, GB, AT, JO, SAC, BG, MA, AO, EB, GS, IN, KL, SS, AD, Ud’A, IC, AE, RG, TB, and CD were involved in data collection, lab processing, and data interpretation of the clinical trials analyzed. NS, LG, JC, TC, and SC decided the list of SNPs to be genotyped. LG, JC, and SC organized and performed the SNP genotyping. NS performed the statistical analysis. NS, LG, and CD wrote the manuscript. All authors have revised, read, and approved the final version of the manuscript.

## Conflict of Interest

JC and LM were employed by the company LGC Genomics. The remaining authors declare that the research was conducted in the absence of any commercial and financial relationships that could be construed as a potential conflict of interest.

## Abbreviations

BF1: dose-efficacy study from Burkina Faso
BF2: safety study from Burkina Faso
DBS: dried blood spots
DP: Dihydroartemisinin-piperaquine
FST: Fluorescence spot test
GAM: safety study from the Gambia
G6PD: Glucose-6-phospate dehydrogenase
Hb: Hemoglobin
Hb0: Hemoglobin levels at day 0
Hb7: Hemoglobin levels at day 7
KEN: dose-efficacy study from Kenya
MAL: safety study from Mali
PQ: Primaquine
RDT: rapid diagnostic test
SNP: Single Nucleotide Polymorphism
UGD: dose-efficacy study from Uganda.
