# Supplementary figures and images for: G6PD Polymorphisms and Hemolysis After Antimalarial Treatment With Low Single-Dose Primaquine: A Pooled Analysis of Six African Clinical Trials
Source: Front Genet. 2021 Apr 9;12:645688. doi: 10.3389/fgene.2021.645688 (PMC8062977; doi:10.3389/fgene.2021.645688)

**A****BF1 – Males**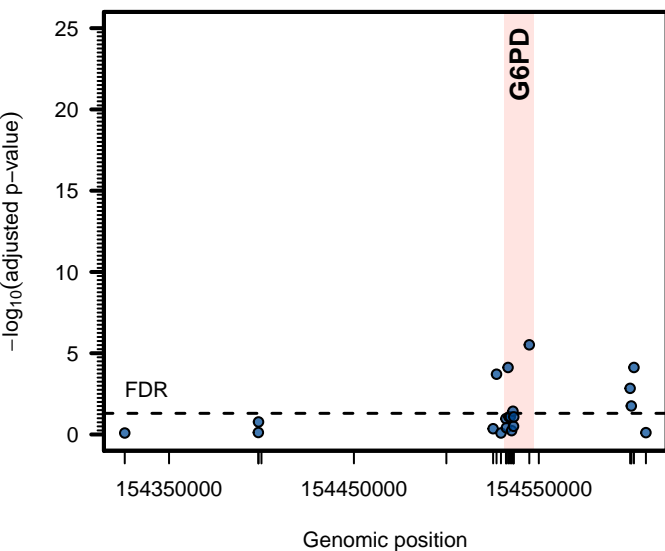**BF2 – Males**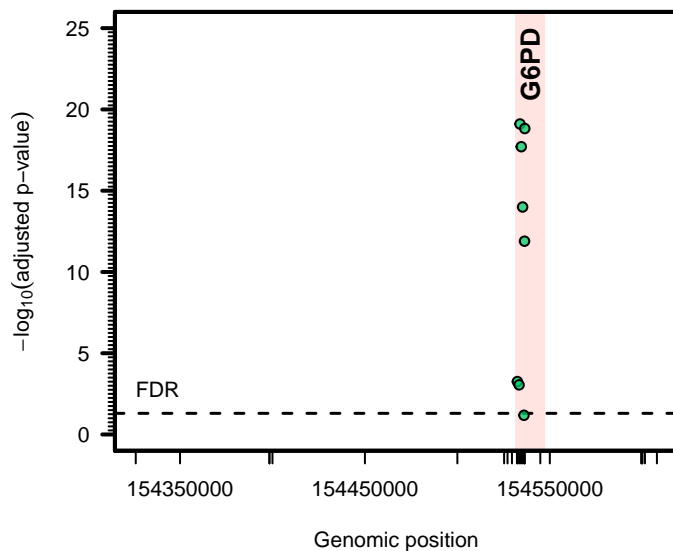**GAM – Males**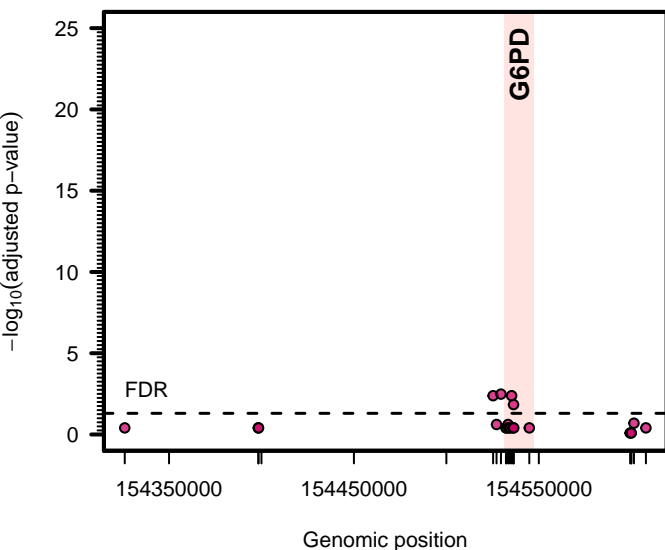**KEN – Males**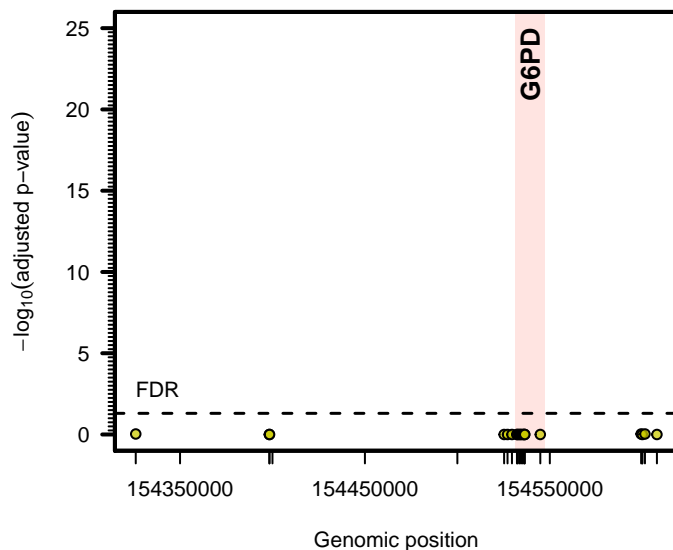**MAL – Males**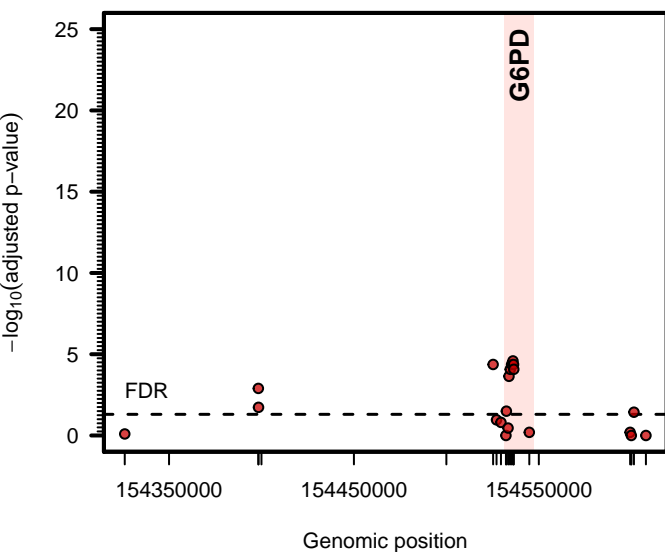**UGD – Males**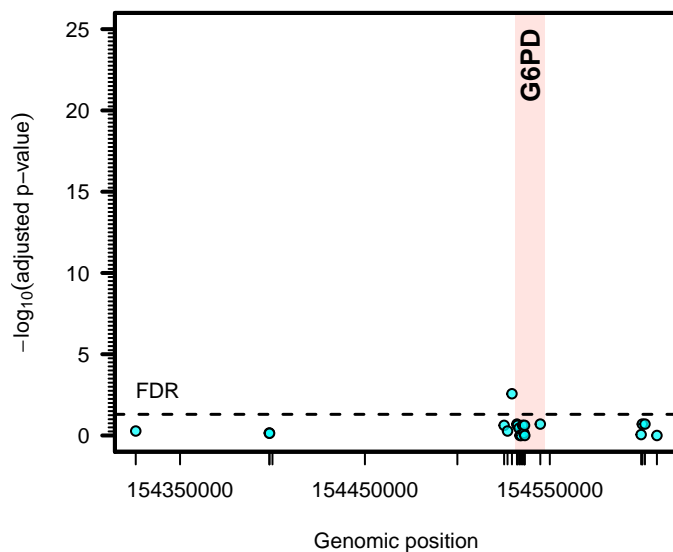

**B****BF1 – Females**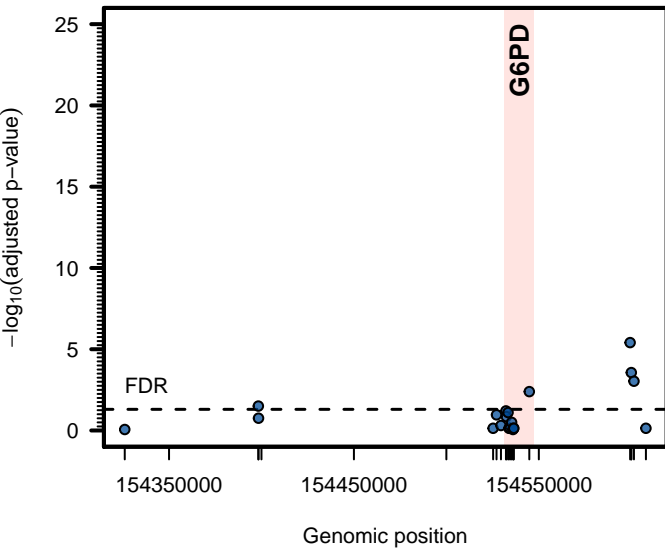**KEN – Females**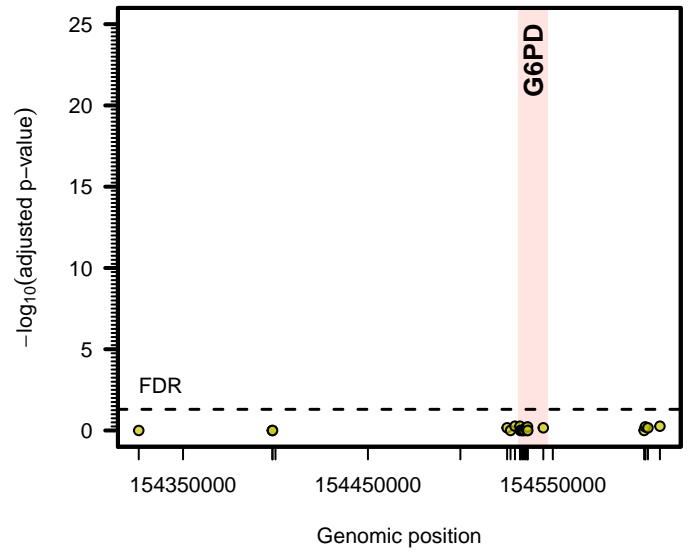**UGD – Females**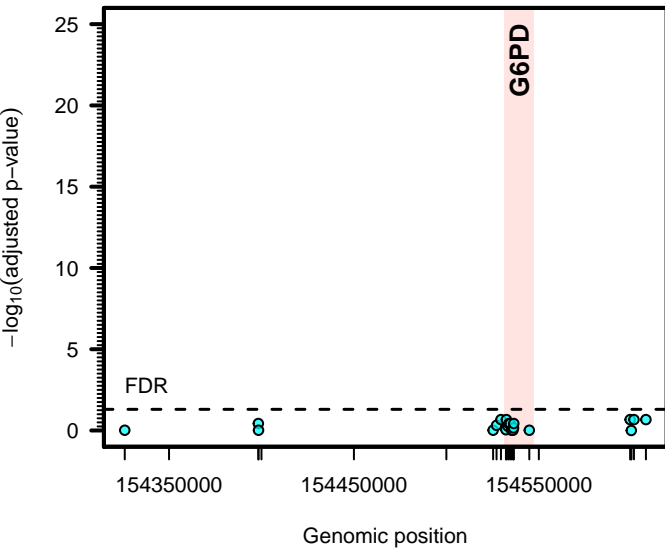

Supplement: Supplementary file 1 [file Data_Sheet_1.PDF]
