# Supplementary material for: G6PD Polymorphisms and Hemolysis After Antimalarial Treatment With Low Single-Dose Primaquine: A Pooled Analysis of Six African Clinical Trials
Source: Front Genet. 2021 Apr 9;12:645688. doi: 10.3389/fgene.2021.645688 (PMC8062977; doi:10.3389/fgene.2021.645688)
Supplement: Supplementary file 2 [file Table_1.DOCX]

**Supplementary table 1**

List of SNP initially selected for this study where the SNPs in bold were included in the final analysis.

| Panel 1: SNPs (KASP, GAM and BF1) | Panel 2: SNPs (KASP, BF2) | Panel 3: SNPs (customised, KEN, MAL and UGD) |
| --- | --- | --- |
| **G6PD_X_b36_153413623** | rs1050757 | **G6PD_X_b36_153413623** |
| **G6PD_X_b36_153426256** | **rs1050828** | **G6PD_X_b36_153426256** |
| **rs1050828** | **rs1050829** | **rs1050828** |
| **rs1050829** | rs111827785 | **rs1050829** |
| **rs12389569** | rs113492957 | **rs12389569** |
| rs2071429 | rs137852318 | rs2071429 |
| rs2230036 | rs137852328 | rs2230036 |
| **rs2230037** | rs1894260 | **rs2230037** |
| **rs2515904** | rs2004651 | **rs2515904** |
| **rs2515905** | rs2071429 | **rs2515905** |
| **rs28470352** | rs2230036 | **rs28470352** |
| **rs4898389** | **rs2230037** | **rs4898389** |
| **rs5986877** | **rs2515904** | **rs5986877** |
| rs5986990 | **rs2515905** | rs5986990 |
| **rs60030796** | rs2515906 | **rs60030796** |
| **rs61042368** | rs35228794 | **rs61042368** |
| rs7053878 | rs5030868 | rs7053878 |
| **rs73573478** | rs5030869 | **rs73573478** |
| **rs762515** | rs5030872 | **rs762515** |
| **rs762516** | rs5986990 | **rs762516** |
| **rs766420** | **rs73573478** | **rs766420** |
| **rs7879049** | **rs762515** | **rs7879049** |
| **rs915941** | **rs762516** | **rs915941** |
| **rs915942** | rs76723693 | **rs915942** |
| rs1050757 |  |  |
| rs111827785 |  |  |
| rs113492957 |  |  |
| rs137852318 |  |  |
| rs1894260 |  |  |
| rs2004651 |  |  |
| rs2515906 |  |  |
| rs35228794 |  |  |
| rs5030869 |  |  |
| rs5030872 |  |  |
