# Supplementary material for: G6PD Polymorphisms and Hemolysis After Antimalarial Treatment With Low Single-Dose Primaquine: A Pooled Analysis of Six African Clinical Trials
Source: Front Genet. 2021 Apr 9;12:645688. doi: 10.3389/fgene.2021.645688 (PMC8062977; doi:10.3389/fgene.2021.645688)
Supplement: Supplementary file 3 [file Table_2.DOCX]

**Supplementary Table 2**

Estimates of a non-genetic linear regression model for describing the Hb log-levels at day 7.

| **Predictor** | | **Estimate (SE)** | **P-value** |
| --- | --- | --- | --- |
| Intercept | | 1.013 (0.065) | < 0.001 |
| Study | |  |  |
|  | BF1 (Reference) | NA | - |
|  | BF2 | -0.021 (0.020) | 0.301 |
|  | The Gambia | -0.010 (0.018) | 0.599 |
|  | Kenya | 0.048 (0.011) | <0.001 |
|  | Mali | -0.009 (0.029) | 0.745 |
|  | Uganda | 0.012 (0.009) | 0.158 |
| Age (per year) | | 0.004 (0.001) | <0.001 |
| PQ dose | |  |  |
|  | 0 (Reference) | - | - |
|  | 0.1-0.4 mg/kg | -0.010 (0.008) | 0.199 |
|  | 0.4-0.75 mg/kg | -0.019 (0.012) | 0.113 |
| Gender | |  |  |
|  | Female (Reference) | - | - |
|  | Male | -0.007 (0.007) | 0.328 |
| log Hb (day 0) | | 0.567 (0.027) | <0.001 |
| Parasitaemia (x 100,000) | | -0.033 (0.005) | <0.001 |
